# Signal pathways and precision therapy of small-cell lung cancer

**DOI:** 10.1038/s41392-022-01013-y

**Published:** 2022-06-15

**Authors:** Min Yuan, Yu Zhao, Hendrik-Tobias Arkenau, Tongnei Lao, Li Chu, Qing Xu

**Affiliations:** 1 Department of Oncology, Shanghai Tenth People’s Hospital, Tongji University, 200072 Shanghai, China; 2 Drug Development Unit, Sarah Cannon Research Institute, London, UK; 3 Department of Oncology, Centro Medico BO CHI, Macao, SAR China; 4 Department of Radiation Oncology, Fudan University Shanghai Cancer Center, 200032 Shanghai, China; 5 Present Address: Department of Oncology, Shanghai Medical College, Fudan University, 200032 Shanghai, China

**Keywords:** Lung cancer, Drug development

## Abstract

Small-cell lung cancer (SCLC) encounters up 15% of all lung cancers, and is characterized by a high rate of proliferation, a tendency for early metastasis and generally poor prognosis. Most of the patients present with distant metastatic disease at the time of clinical diagnosis, and only one-third are eligible for potentially curative treatment. Recently, investigations into the genomic make-up of SCLC show extensive chromosomal rearrangements, high mutational burden and loss-of-function mutations of several tumor suppressor genes. Although the clinical development of new treatments for SCLC has been limited in recent years, a better understanding of oncogenic driver alterations has found potential novel targets that might be suitable for therapeutic approaches. Currently, there are six types of potential treatable signaling pathways in SCLC, including signaling pathways targeting the cell cycle and DNA repair, tumor development, cell metabolism, epigenetic regulation, tumor immunity and angiogenesis. At this point, however, there is still a lack of understanding of their role in SCLC tumor biology and the promotion of cancer growth. Importantly optimizing drug targets, improving drug pharmacology, and identifying potential biomarkers are the main focus and further efforts are required to recognize patients who benefit most from novel therapies in development. This review will focus on the current learning on the signaling pathways, the status of immunotherapy, and targeted therapy in SCLC.

## Introduction

Lung cancer (LC) is the major cause of cancer mortality worldwide and has the highest morbidity among all cancers. It is estimated that approximately 228,820 new cases of lung cancer will be diagnosed in the United States (US) in 2020 and up to 135,720 patients will die of the disease.^1^ Overall 15% of LC patients are diagnosed with small-cell lung cancer (SCLC).^2^ Almost all cases of SCLC are associated with tobacco smoking. Classical features of SCLC are rapid disease progression and a tendency to the early development of widespread metastases. As a result, nearly 80-85% of patients present with extensive-stage small-cell lung cancer (ES-SCLC) at the time of diagnosis.^3^ SCLC usually shows high sensitivity to initial chemotherapy and radiotherapy. For several decades, the standard of care for patients with ES-SCLC were chemotherapy regimens based on platinum drug combinations and have shown survival benefit. However, despite initial good clinical response to treatment, the median survival rarely exceeds 1 year.^4^ Most patients with ES-SCLC eventually progress and succumb to recurrent disease^5^ with only 10–20% surviving beyond 2 years.^6^

**Fig. 1:**
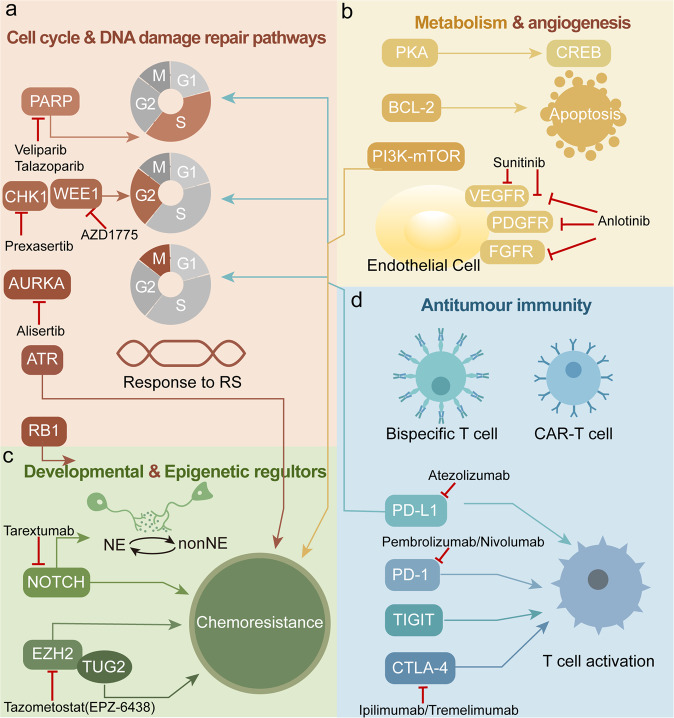
Six main representative therapeutically tractable targets in small-cell lung cancer (SCLC). **a** Cell cycle and DNA damage repair pathways. PARP poly (ADP)-ribose polymerase, AURKA Aurora A kinase, CHK1 checkpoint kinase 1, ATR Ataxia telangiectasia and rad3 related, RS replication stress. **b** Metabolism and angiogenesis signaling pathways. PKA protein kinase A. **c** Developmental and epigenetic regulators. NE neuroendocrine, TUG taurine upregulated gene1. **d** Antitumour immunity. PD-1 programmed death-1, PD-L1 programmed death ligand-1, CTLA-4 cytotoxic T-lymphocyte-associated antigen 4, TIGIT T-cell immunoreceptor with immunoglobulin and ITIM domains

In the past few years, genomic analysis of SCLC has shown a high mutational burden rate and extensive chromosomal rearrangements, almost always involving the inactivating mutations of TP53 and RB1, and often accompanied by the MYC oncogene expression which resulting in rapid cell proliferation and DNA replication stress.^6,7^ These genetic alterations result in genomic instability and increased tumor-associated antigens (TAAs) presence.^8^ Furthermore, SCLC is often associated with high tumor mutational burden (TMB),^9^ favoring the use of immunotherapy combined with chemotherapy in the first-line setting or single-agent immunotherapy of subsequent treatment lines.^10–12^ In addition, mutations in MLL, PTEN, PI3KCA, SLIT2, Notch genes, EPHA7, and amplification of FGFR1, MYC, and SOX2, have also been reported.^13^ Based on the heterogenous alterations of multiple genes and pathways, SCLC can be classified into different molecular subtypes potentially opening opportunities to target those with subsequent better response and outcome. In addition to new drugs and drug classes with novel mechanisms of action, there is a role to re-visit multiple drugs which have previously failed to demonstrate clinical benefit in SCLC.^14^ A large number of recruiting and ongoing clinical trials are evaluating new targeted drugs for the systemic therapy of SCLC (Fig. 1 and Table 1).

**Table 1:** Select recent clinical trials of novel targeted therapies in small-cell lung cancer

Target	Agent	Clinical setting	Phase	NCT numbers	Status	Estimated completion date
PARP	Olaparib	Olaparib plus temozolomide	I/II	NCT02446704	Active	December 2023
CHK1	SRA-737	SRA-737 in combination with gemcitabine plus cisplatin (versus in combination with low-dose gemcitabine)	I/II	NCT02797977	Completed	April 2020
Aurora A kinase	Alisertib	Paclitaxel with or without alisertib	II	NCT02038647	Completed	July 10, 2017
ATR kinase	M6620	Topotecan with or without M6620 in relapsed SCLC	II	NCT03896503	Suspended	September 1, 2023
WEE1	AZD1775	AZD1775 plus carboplatin as 1 arm of a multiarm phase 2 study of novel combinations in platinum-refractory SCLC	II	NCT02937818	Active	December 30, 2022
Bcl-2/mTOR	Navitoclax and Vistusertib	Relapsed SCLC and other solid tumors	I/II	NCT03366103	Active	August 31, 2022
IDH inhibitors	ivosidenib (AG-120)	solid tumors	I	NCT02073994	Active	June 2022
DLL3	AMG 119	Relapsed/Refractory Small-Cell Lung Cancer	I	NCT03392064	Active	January 13, 2026
Hedgehog pathway inhibitor	Vismodegib (GDC-0449)	Cisplatin and etoposide in combination with either Hedgehog inhibitor GDC-0449 or IGF-1R MOAB IMC-A12 for patients with extensive-stage small-cell lung cancer	II	NCT00887159	Completed	January 5, 2021
LSD1 inhibitor	Bomedemstat (IMG-7289)	Bomedemstat and maintenance immunotherapy for treatment of newly diagnosed extensive-stage small-cell lung cancer	I/II	NCT05191797	Not yet recruiting	January 15, 2026
EZH1/2	DS-3201b	DS-3201b plus irinotecan in recurrent SCLC	I/II	NCT03879798	Recruiting	March 2023
VEGFR, PDGFR and FGFR	Anlotinib	Anlotinib plus platinum etoposide in first-line of extensive-stage small-cell lung cancer	II	NCT04675697	Recruiting	January 31, 2021

## Signaling pathways targeting the cell cycle and DNA repair

### Poly (ADP-ribose) polymerase (PARP)

PARP enzymes, activated by binding single-strand DNA breaks (SSB), belong to a family of DNA Damage Repair (DDR) proteins, which has a critical role in recognizing and repairing DNA breaks, transcriptional regulation, and chromatin remodeling. The function of PARP involves poly-ADP ribosylation (PARylation) of diverse substrates and proteins recruitment to mediate DNA repair. Poly-(ADP)-ribose glycohydrolase (PARG) and other enzymes subsequently metabolize PAR groups, which is critical to effective DNA repair. Including mutations, chromosomal translocations, deletions, and amplification, replication aberrancies can lead to cell death, senescence, or malignant transformation if double-strand breaks (DSBs) are not correctly repaired.^15^ PARP inhibitors have been proven to overcome resistance to chemotherapy or other therapeutic methods. Overexpression of PARP1 indicates good therapeutic response for inhibiting PARP in SCLC.^16^ Studies have shown two main mechanisms of PARP inhibitors to exert their effect: trapping the enzyme to the SSBs and preventing PARylation and binding of PARP to DNA.^17^ Many PARP inhibitors, such as olaparib, talazoparib (BMN-673), pamiparib (BGB-290), and veliparib (ABT-888) are undergoing active clinical investigation in SCLC.^18–22^

### Checkpoint kinase 1 (CHK1)

Previous studies found that frequent loss of RB1 in SCLC leads to the loss of E2F1 inhibition, which in turn is related to increased expression of several key mediators of DDR, such as PARP1 and in particular the CHK1 protein.^16^ CHK1 is an essential serine-threonine kinase and the primary mediator of DNA damage-dependent cell cycle arrest.^23–26^ A biomarker study for example has shown significantly higher CHK1 protein expression in SCLC compared to normal lung tissue.^27^ In this context, CHK1 inhibitors can increase DNA damaging effects and early preclinical data indicate CHK1 to be a promising target for SCLC, especially in the subset of tumors with cMYC protein overexpression.^27^

A second-generation CHK1 inhibitor, Prexasertib (LY2606368), has been investigated alone or combined with chemotherapy in platinum-sensitive and resistant SCLC models.^28^ Prexasertib has shown significant single-agent activity in a panel of 39 SCLC cell lines from human and murine, especially in chemotherapy-resistant models.^27^ Thus, it reinforced the cisplatin effect in both chemotherapy-resistant and -sensitive models, indicating the potential clinical relevance. In addition, inhibition of CHK1 did overcome the resistance to PARP inhibitor in preclinical studies.^29,30^ Another CHK1 inhibitor in clinical development is SRA-737, an oral small-molecule CHK1 inhibitor. Several clinical trials are ongoing for testing SRA-737 as monotherapy or combined with gemcitabine in patients with a variety of solid tumors including SCLC (NCT0279964, NCT02797977).^31^ Furthermore, a recent study showed that targeting the two DDR proteins, PARP or CHK1, remarkably increased expression of PD-L1. The emerging immunomodulatory functions of DDR inhibitors have not been previously identified, but suggest that the combination of PARP or CHK1 inhibitors with immune checkpoint inhibitors may improve the therapeutic index in patients with SCLC. In addition, the study supported a novel role of the innate immune STING pathway in DDR-mediated antitumor immune response in SCLC.^32^

### Aurora A kinase (AURKA)

Aurora A plays a critical role in mitosis and belongs to a member of the Aurora protein family. In tumor cells, the gene is commonly amplified, which leads to an overexpression of Aurora A. Chromosome alignment, segregation, centrosome maturation and cytokinesis are critical mitotic events and are controlled by Aurora A.^33^ In this context, a recent study showed that the Aurora A inhibitor could inhibit the proliferation and induce G2/M phase arrest of SCLC cells.^34^ It seems that oncogenesis can also be activated by its overexpression by causing genomic instability. Thus, overexpression of Aurora A is associated with the worse outcome in several tumor entities.^35^ In a randomized phase II study of paclitaxel plus the selective Aurora Kinase A inhibitor, Alisertib (MLN8237; Takeda), versus paclitaxel plus placebo as the second-line treatment for SCLC, achieved a considerable 22% objective response rate (ORR).^36^ Despite the promising response rate, this combination was associated with an increased risk of serious adverse drug events.

### Ataxia telangiectasia and rad3 related (ATR)

Replication stress (RS) is a common characteristic with activated oncogenes or inactivated cancer suppressor genes which in turn speed up the rate of S-phase entry and subsequently impair the orderly DNA replication process in cancers.^37^ In SCLC, the high degree of genomic instability, frequent amplification/overexpression of oncogenes and the high expression of lineage transcription factors contribute to RS.^38,39^ Ataxia telangiectasia mutated and rad3 related (ATR) is the primary responder to RS and inhibition of ATR could exacerbate cell death and enhance chemotherapy activity in particular of topoisomerase I (TOP1).^40^ For example, a preclinical study showed ATR had an important role in the clearance of TOP1 DNA-protein crosslinks (TOP1-DPCs) and repair of DNA damage, mediated by TOP1. ATR inhibition enhanced TOP1-mediated DNA damage by topotecan. It demonstrated that DNA replication stress as a therapeutic vulnerability of SCLC, higher neuroendocrine differentiation and enhanced RS had the more sensitivity to ATR and TOP1 inhibition, which contributed durable tumor release in platinum-resistant patients. As such, for platinum-resistant SCLC, which acquired resistance through upregulation of DDR pathways, ATR inhibitor might sensitize it.^41^

In a phase I trial, the first-in-class, ATP-competitive inhibitor of ATR (M6620) was combined with topotecan in SCLC patients who relapsed after at least one prior lines of systemic treatment. Plasma pharmacokinetics of topotecan and M6620 appeared consistent with the individual agents, suggesting the absence of drug-drug interactions. Most adverse events were attributable to topotecan. Three patients with SCLC achieved durable clinical benefit (one PR and two prolonged SD). The median PFS of patients with SCLC was 10.2 months, and the 6-month PFS probability was 60.0%.^42^

### WEE1

WEE1 is a G2 checkpoint kinase, and an important part of the G2/M checkpoint. It prevents entry into mitosis when cellular DNA is damaged, and frequently expressed at high levels in various cancer types.^23^ Inhibition of WEE1 has shown promise as an antitumor strategy, especially in cancers with inactivated TP53.^24^ The WEE1 inhibitor AZD1775 has been analyzed in a phase Ib trial for patients with advanced solid tumors, including SCLC (NCT02482311).^43^

## Signaling pathways targeting metabolism

### Protein kinase A (PKA)

Recent studies showed that PKA also played an important role in SCLC. PKA is a tetramer with two regulatory and two catalytic subunits. Unbinding the regulatory subunits to cyclic AMP (cAMP) activates the kinase (PKA-Ca) to promote and maintain SCLC development. PKA-Ca is found to be the dominant catalytic subunit in SCLC and 17.5% of SCLC have been reported to have a PKA/CREB (cAMP response element-binding protein) pathway activation. A preclinical study showed that a reduction in PKA-Ca activity can block SCLC survival in mouse models.^44^ Moreover, Rb/Trp53/Rbl2 triple-knockout SCLC tumors also showed higher PKA expression compared to normal mouse lung tissue.^45^ Previous studies have also investigated potentially functional interactions between PKA and phosphatase 2A (PP2A).^46^ Inhibition of PKA or regulation of the other enzymes in the PKA signaling pathway, for example, CREB,^47^ may have antitumor effects in SCLC. In this context metformin, an inhibitor of apoptosis proteins, has shown antitumor activities in several types of cancer beyond its activity as a first-line antidiabetic drug. A recent study showed that metformin could suppress PKA activity and induce the activation of its downstream effectors, such as synthase kinase 3β (GSK-3β), resulting in SCLC cell death by AMPK/PKA/GSK-3β axis mediated surviving degradation.^48^

### PI3K/AKT/mTOR pathway

The PI3K/AKT/mTOR pathway is associated with the proliferation, migration, and survival of SCLC.^49^ Therefore, patients can be stratified by potential therapeutic targets using a sequencing-based comprehensive analysis. SCLC is well known with poor prognosis, and few targeted drugs have shown clinical efficacy over an extended period. Genetic alterations of the PI3K/AKT/mTOR pathway were considered to be a potential therapeutic strategy in SCLC. The PI3K-AKT-mTOR pathway is also an important metabolic regulator.^50,51^ The role of PI3K-dependent kinase 1 and mTORC2 in the phosphorylation of specific serine/threonine residues is vital for AKT activation.^52^ mTOR activation can regulate the expression of metabolic gene and contribute the metabolic processes.^53^

P53 can be reactivated by Nutlin-3 interacting specifically with the complex of p53-Mdm2 and promote cancer apoptosis. Several possible mechanisms for the resistance including insensitivity of cancer cells are relative to it. Potential master regulators could be identified by analyzing the signal transduction network upstream of these transcription factors. The primary regulators were responsible for maintaining low sensitivity of cancer cells to Nutlin-3, which had an important role in activating PI3K pathway. Those cell lines, Nutlin-3 insensitive, showed the remarkable sensitivity to the dual inhibitor of mTOR-PI3K.^54^ In addition, preclinical study found chemoresistance could be reversed by the PI3K/AKT/SOX2 signaling pathway in SCLC.^55^ More and more PI3K and mTORC1/2 inhibitors, single or dual, were designed for the patients in phase I study.^56^ Molecular alterations in the PI3K/AKT/mTOR signaling pathway are considered to be a precise therapeutic priority in SCLC for the selection of patients, who are potentially sensitive to PI3K/AKT/mTOR inhibitors.

### BCL-2

The Bcl-2 proteins family is mainly involved in mediating cell apoptosis. The elevated level of Bcl-2 is observed in SCLC and is associated with poor prognosis.^57^ As a critical apoptosis regulator, Bcl-2 is widely expressed in SCLC, and multiple studies show synergy of inhibiting the Bcl-2 and PI3K/mTOR pathways in SCLC.^58^ Of note, a biomarker-based approach showed in a preclinical context encouraging results of Venetoclax in high Bcl-2 expressing SCLC cells lines.^59^ Unfortunately, Obatoclax (a first-generation Bcl-2 inhibitor) and AT-101 (a small-molecule Bcl-2 inhibitor) showed a lack of clinical benefit in phase II studies.^60,61^ It remains speculative whether these molecules simply showed insufficient potency against Bcl-2 or whether the pathway is not as critical as initially thought. Currently, several clinical studies investigate the role of other Bcl-2 inhibitors including, ABT-263.^62^

### MYC family genes

Amplifications in the MYC family are the second most common genetic alterations in SCLC beyond TP53 and RB1. Amplification and/or overexpression of MYC family genes are observed in about 20% of tumors, including MYC, MYCL, and MYCN.^63^ MYC amplification is found to drive genomic instability and replication stress (RS). Activation of genes of the MYC family can sensitize tumors to Aurora kinase inhibitors^64^ and first preclinical studies in cMYC positive tumors achieved remarkably better progression-free survival (PFS) compared to those with cMYC-negative tumors when alisertib (Aurora Kinase A inhibitor) was combined with paclitaxel, but OS was not improved.^65^ Moreover, Aurora kinase genes, knockdown by short hairpin RNA (shRNA), or small-molecule Aurora kinase inhibitors could lead to apoptosis of MYC-amplified cell lines. A preclinical study has proved the sensitivity to Aurora kinase inhibitors in MYC-driven SCLC, together with chemotherapy.^66^ Similarly, in another preclinical study, amplification or activation of any one of the three MYC family genes could predict the effects to Aurora kinase inhibitor.^67^

Studies have reported that MYCN and MYCL play an important part in the metabolism and drug resistance of SCLC. MYC-driven SCLC highly relies on arginine-regulated metabolic pathways, such as biosynthesis of polyamine and activation of mTOR.^68^ It was found that deubiquitinase USP7 is a druggable synthetic fragility in MYCN-associated SCLC and MYCN overexpression is the driver of SCLC chemoresistance.^69^

### Glucose metabolism pathway

Glycolytic enzymes and transporters of the glucose metabolic pathway are reported to be overexpressed in multiple cancers, including lung cancer.^69^ Different transcription factors and signal pathways are vital drivers of glucose metabolism.^70,71^ Inhibiting these pathways or downstream targets (glycolytic enzymes, glucose transporters, mitochondrial pore) is relevant and has attracted multiple drug development groups to develop specific inhibitors. For example, inhibitors targeting glycolytic transporters and enzymes are currently in pre- and clinical development, and most importantly some have reached approval status, i.e., IDH inhibitors. Challenges in developing metabolism-based therapies are that they include exactly the same metabolic pathways for cell survival and division, and the treatment may face the difficulty of non-specific toxicity.^72–74^ The glycolytic pathway is critical for the proliferation and functional activity of immune cells.^75^ Therefore, activated immune cells may cause immune suppression because they are susceptible to glycolysis inhibition.^76^ Small molecules (such as CB-839, compound 968 or BPTES) simultaneously inhibit the glutaminolysis pathway may be a valuable therapeutic strategy.^77^

## Signaling pathways targeting development

Cancer stem cells (CSCs) share many characteristics of embryonic stem cells and demonstrate the ability to continuously activate one or more signaling pathways involved in the development, including the Notch and Hedgehog pathways, which may be elevated in small-cell lung cancer.^78,79^

### NOTCH pathway

Research found that SCLC cells showed a significant growth arrest with active Notch1/2, attributing to activation of p21 and arrest of G1 cell cycle.^6^ Notch knockdown may lead to an increase in cell proliferation, validating the antitumor effect in SCLC cells.^80^ Furthermore, the Notch pathway had an identifying role in regulating neuroendocrine gene expression in SCLC by specimens’ genomic analysis. Notch signaling was demonstrated to drive plasticity from neuroendocrine (NE) to nonneuroendocrine (nonNE) phenotypes.^81^ It was investigated that inactivating Notch mutation could improve nonNE tumor cells or precursors to neuroendocrine differentiation.^82^ Notch-ASCL1 signaling contributes to drive and maintain the phenotype of small cancer cells. When Notch active, achaete-scute homolog 1 (ASCL1) may have cooperation with biallelic RB/p53 loss, and may lead to anticancer-caused secondary SCLC arising from NSCLC^83^. Tarextumab (OMP-59R5) is a fully human monoclonal IgG2 antibody which binds and selectively inhibits signaling via both Notch2 and Notch3. It was investigated in SCLC allografts and patient-derived tumor xenografts (PDX) firstly, and then clinical practice,^84,85^ in combination with chemotherapy. In theory, synergistic effect can be achieved by combination Tarextumab with chemotherapy, both killing the most chemosensitive neuroendocrine cancer cells and inhibiting the Notch pathway, the switch to a nonneuroendocrine phenotype. The study showed three drugs regimen (tarextumab, carboplatin, and irinotecan) performed better in tumor suppression than any of them alone in both models. Excitingly, tarextumab was found to have an important role in delaying chemoresistance process in these PDX models.

A phase Ib/II study (PINNACLE) of tarextumab in combination with chemotherapy explored the pharmacokinetics (PK), pharmacodynamics (PD), and preliminary efficacy of Tarextumab combined with EP in chemo-naive ED-SCLC. The phase I study conducted on 27 patients showed tarextumab was well tolerated in combination with chemotherapy, However, PFS was 5.6 months in the tarextumab arm and 5.5 months in the placebo arm respectively, with no significant difference. The ORR and OS were also extremely similar between the two arms.^86^

Delta-like ligand 3 (DLL3) is a Notch pathway inhibitory ligand, and highly upregulated and aberrantly expressed in SCLC and other high-grade neuroendocrine tumors.^87^ Notch signaling is downregulated and inhibited by DLL3 expression during the growth of neuroendocrine tumors.^88^ In preclinical models, expression of DLL3 promotes invasion and migration of SCLC.^89^ Rovalpituzumab tesirine is an antibody-drug conjugates (ADC) targeting DLL3, clinical studies of a combination therapy of rovalpituzumab tesirine and immune checkpoint inhibitor therapy are expected to better understand the therapeutic role of this target and may eventually offer patients a more valid treatment regimen.^90^

### Hedgehog signaling

The Hedgehog signaling pathway regulates the proliferation and differentiation during embryonic development and is involved in early lung development through the epithelial mesenchymal interaction.^91,92^ It is reported that the expression of signaling molecules in sonic Hedgehog pathway are upregulated in SCLC and play an important part in the development and proliferation of SCLC.^93^ Preclinical studies found that inhibiting Hedgehog may delay or prevent disease recurrence in patients after chemotherapy.^94^ The selective Hedgehog pathway inhibitor Vismodegib (GDC-0449) can block Hedgehog signaling by binding to SMO and inhibiting the activation of Hedgehog target genes.^95^ A multicenter, open-label study was designed for patients with ED-SCLC. In total, 152 patients were treated with etoposide and cisplatin or combined with vismodegib. However, the results indicated that vismodegib did not improve PFS or OS significantly.^96^

## Signaling pathways targeting epigenetic regulation

### Histone modifiers

Epigenetic variabilities contribute to the development of cancer directly.^97^ Recurrent genetic alterations in histone-modifying genes appear to be a hallmark of SCLC.^13^ The transcription enhancer CREB binding protein gene (CREBBP) is one of the most commonly mutated genes in SCLC.^98^ CREBBP has been considered as a predictive biomarker for volasertib (a polo-like kinase 1 inhibitor) according to the outcomes of genome-wide screening and genetic analysis.^99^ These studies also indicated that the effects of volasertib and deacetylase inhibitors depended on the mutational status of CREBBP or histone acetylation, confirming the importance of histone acetylation in developing therapies targeting epigenetic alterations in SCLC. E1A binding protein p300 gene (EP300) is another partner of CREBBP, which is inactivated or mutated along with CREBBP. EP300 also has an intrinsic histone acetyltransferase (HAT) activity, which can regulate chromatin remodeling and access to other transcription factors, and acts as a key regulator of biological functions such as homeostasis, cell growth, and embryonic development.^100,101^

The bromodomain and extraterminal (BET) family proteins play a pivotal role in transcriptional regulation and could interact with different chromatin modifiers, such as HATs and HDACs.^102^ The BET family members are highly expressed in SCLC cell lines with amplification of MYC family. Several early clinical trials have been designed to explore the preliminary effectiveness and safety of BET inhibitors in SCLC patients.^103^ Inhibition of the BET family member BRD4 or the cyclin-dependent kinase 7 (CDK7) may provide potential therapeutic targets by inhibiting expression of MYC family members.^28^ Lysine demethylase 1A (LSD1) encodes a histone modification enzyme which is overexpressed in SCLC and many other malignant tumors.^104^ The LSD1 inhibition provides a promising new therapeutic target for the SCLC patients. Iadademstat (ORY-1001) is a highly selective LSD1 inhibitor, which mediates LSD1 inhibition and downregulates the expression of ASCL1, thereby reducing the tumorigenesis of SCLC.^105^ Another selective, oral LSD1 inhibitor (GSK2879552) has also shown antitumor properties in both SCLC cell lines and tumor models.^106^ Overall, the preclinical studies of LSD1 inhibitors in SCLC suggest that it has the potential to develop epigenetic therapies for SCLC.

### Enhancer of zeste homolog 2 (EZH2)

The histone methyltransferase EZH2 is an oncogene that is highly expressed in SCLC,^107^ and has an important role in both SCLC chemoresistance and immune escape.^108,109^ In addition, overexpression of EZH2 is related to tumorigenesis, metastasis, cancer progression, and poor prognosis.^110^ Inhibition of EZH2 combined with chemotherapy is currently being investigated in a phase I/II clinical trial of recurrent SCLC patients.^111^ As a histone methyltransferase component of the polycomb repressive complex 2 (PRC2) and a known target of E2F, EZH2 has been implicated in dysregulation of DNA methylation through its effects on histone methylation. In SCLC, EZH2 was found to be activated by gene copy loss and function mutations loss in RB1, by which the E2F repressor pRB is encoded.^107^ EZH2 has also an important role of promoting cell growth and chemoresistance in SCLC. Taurine upregulated gene1 (TUG1) regulates the expression of LIMK2b (a splice variation of LIM-kinase 2) by means of binding to the enhancer of EZH2, and mediate chemoresistance in SCLC.^112^ Furthermore, new findings reveal that EZH2 promote DDB2 stabilization and NER in a non-catalytic and PRC2-independent way and reverse cisplatin resistance in SCLC, suggesting a rationale for overcoming cisplatin resistance in SCLC by targeting EZH2 beyond its catalytic activity.^113^

## Signaling pathways targeting tumor immunity

### Cytotoxic T-lymphocyte-associated antigen 4 (CTLA-4)

Central to peripheral tolerance process are the CTLA-4 and PD-1 immune checkpoint pathways.^114^ CTLA-4 is considered the “leader” of the immune checkpoint inhibitors (ICIs). It can prevent autoreactive T lymphocytes at the initial activation phase of naive T-cell.^115^ CTLA-4 is a homolog of CD28, and has a higher binding affinity to B7. However, the combination of CTLA-4 and B7 does not transduce a stimulatory signal, which is different from CD28. Therefore, the costimulatory signal normally provided by CD28:B7 binding can be prevented by this competitive binding.^116,117^ The relative amount of CTLA-4:B7 binding versus CD28:B7 binding can determine whether a T-cell will be activated or remains anergic.^118,119^ CTLA-4 is also related to other aspects of immune regulation. Regulatory T cells (Tregs) can express CTLA-4 constitutively, which is considered to be dominant for their suppressive functions.^120^ In tumor models, suppressive functions of Tregs can be impaired by genetic CTLA-4 deficiency.^121^ One of the mechanisms of Tregs controlling effector T cells is downregulating B7 ligands on antigen-presenting cells (APCs), and then reducing CD28 costimulation.^122^

Ipilimumab is the first human IgG1 monoclonal antibody targeting the CTLA-4 antigen.^123^ However, it has generally shown limited activity in patients with ES-SCLC. A phase II study investigated the evaluation of ipilimumab added to standard chemotherapy.^124^ In this study, a total of 130 patients were enrolled, and though ipilimumab improved irPFS compared to the chemotherapy, there was no statistical difference of PFS and OS. In a follow-up phase III study, patients with ES-SCLC received chemotherapy and placebo or ipilimumab, and then followed by maintenance ipilimumab.^125^ Patients demonstrated a median OS of 10.9 months in the control arm, compared to 11.0 months in the ipilimumab group. There was no statistical difference of median OS between the two groups.

### PD-1/PD-L1 pathway

Programmed death-1 (PD-1) is expressed on the surface of activated T and B cells, and also a negative regulator of immunity that limits the function of T and B cells.^126^ Signaling through PD-1 pathway can limit the function of T cells, including interferon-γ (IFN-γ) production, proliferation, and increasing T-cell apoptosis.^127,128^ Programmed death ligand-1 (PD-L1) is one of the receptors in the immunoglobulin superfamily, which can regulate T-cell antigen receptor signaling negatively by interacting with PD-1 and play an important part in maintaining self-tolerance. Effective blockade of PD-1 and PD-L1 interaction could provide a promising strategy of immunotherapy for tumors expressing PD-L1.^129^ Expression of PD-L1 on tumor and immune cells has been selected as an important biomarker predicting the therapeutic response to anti-PD-1/PD-L1 treatment in patients with SCLC.^130^

**Fig. 2:**
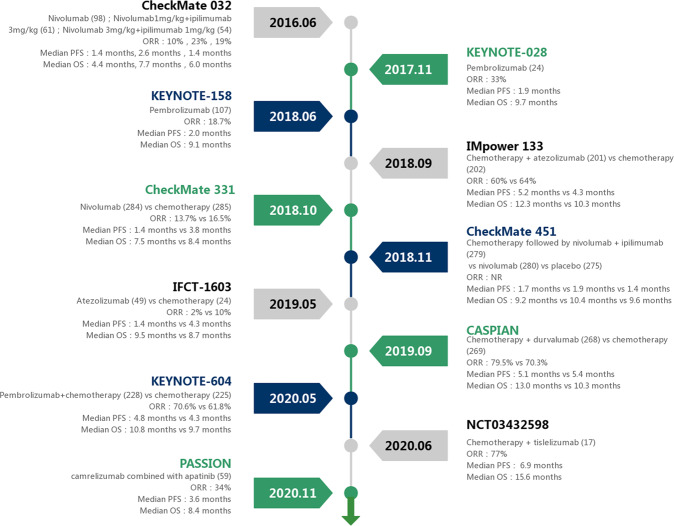
Timeline of key studies of immune checkpoint inhibitors in SCLC. This timeline describes some key studies using immune checkpoint inhibitors in patients with SCLC. NR not reported, ORR objective response rate, PFS progression-free survival, OS overall survival

SCLC is an immunogenic disease due to the increased incidence of autoimmune paraneoplastic phenomena and therefore may be a good candidate for ICIs treatment. Interestingly, the outcomes in patients with SCLC are associated with neurological paraneoplastic syndromes.^131^ It has been demonstrated that adding an ICI to the first-line chemotherapy in metastatic SCLC patients can achieve significant benefits witnessed in multiple randomized phase III studies.^11,12^ Overall, immune checkpoint inhibition doubled the 2-year survival rate from 11 to 22%.^132^ At present, there are still many clinical studies in progress (Fig. 2 and Table 2). Searching for the tumor and patient characteristics related to immunotherapy response is an active area of interest.

**Table 2:** Checkpoint inhibitors combinations with a chemotherapy in the clinical trial

Checkpoint inhibitors	Patients enrolled	Clinical setting	Phase	NCT numbers	Status	Estimated completion date
Ipilimumab and Nivolumab	40	Recurrent extensive-stage small-cell lung cancer after receiving platinum-based chemotherapy	II	NCT03670056	Recruiting	June 2022
Durvalumab andCeralasertib	30	Treatment-naive patients with extensive-stage small-cell lung cancer	II	NCT04699838	Not yet recruiting	February 2024
Durvalumab	63	Olaparib and durvalumab with carboplatin, etoposide, and/or radiation therapy for the treatment of extensive-stage small-cell lung cancer, PRIOTrial	I/II	NCT04728230	Recruiting	July 2022
Sintlimab	40	Maintenance therapy in patients with extensive small-cell lung cancer	II	NCT03983759	Recruiting	May 2021
Atezolizumab	138	Addition of radiation therapy to the usual immune therapy treatment for extensive-stage small-cell lung cancer, the RAPTOR trial	II/III	NCT04402788	Recruiting	April 2027
Durvalumab	43	Treatment of relapsed or refractory small-cell lung cancer	II	NCT04607954	Recruiting	November 2024
Camrelizumab	45	Combined with apatinib, etoposide and cisplatin treat small-cell lung cancer	III	NCT04490421	Recruiting	February 2022
Nivolumab and Ipilimumab	80	Concurrent or sequential immunotherapy and radiation therapy in patients with metastatic lung cancer	I	NCT03223155	Recruiting	December 2024
Atezolizumab	212	After concurrent chemoradiotherapy versus chemoradiotherapy alone in limited-disease small-cell lung cancer	II	NCT03540420	Recruiting	December 2026
Atezolizuma	70	Patients with ES-SCLC and ECOG PS=2 receiving carboplatin etoposide	II	NCT04221529	Recruiting	June 2024
Durvalumab	46	Thoracic radiotherapy plus maintenance durvalumab after first-line carboplatin and etoposide plus durvalumab in extensive-stage disease small-cell lung cancer (ED-SCLC).	II	NCT04472949	Not yet recruiting	December 2027
Atezolizumab	94	Testing maintenance therapy for small-cell lung cancer in patients with SLFN11-positive biomarker	II	NCT04334941	Recruiting	November 2025

#### (1) Atezolizumab

Atezolizumab is the first anti-PD-L1 antibody approved for the first-line treatment combined with carboplatin and etoposide of ES-SCLC worldwide. The approval was based on the primary data from a multinational phase III trial (IMpower133) in treatment-naive ES-SCLC patients. In this trial, atezolizumab was added as an induction regimen to chemotherapy of carboplatin and etoposide and was followed as a maintenance regimen. Compared with chemotherapy alone, it provided a median PFS (5.2 months) benefit of ≈1 month and a median OS (12.3 months) benefit of 2 months. Notably, subgroup analyses showed that patients benefited from atezolizumab, regardless of the PD-L1 or bTMB expression status. However, the overall expression of PD-L1 in SCLC was relatively low in most studies compared with NSCLC.^133^ In addition, the lack of correlation between efficacy and PD-L1 expression in this trial is not only consistent with the results of an earlier phase I clinical trial of atezolizumab monotherapy in patients with relapsed or refractory SCLC^134^ but also the other completed studies of ICIs in patients with untreated ES-SCLC.^135^ Therefore, the use of atezolizumab in patients with ES-SCLC does not mandate PD-L1 testing and expression.^136,137^

#### (2) Durvalumab

Durvalumab is a humanized, selective, and high-affinity monoclonal IgG1 antibody. In the CASPIAN study, durvalumab plus chemotherapy as the first-line treatment significantly improved the OS in patients with ES-SCLC, compared with platinum etoposide alone.^138^ This was an open-label, sponsor-blind, randomized, controlled phase III clinical trial in 209 cancer centers and 23 countries worldwide. Patients received either standard platinum-based chemotherapy or combined with durvalumab 1500 mg or durvalumab plus tremelimumab 75 mg every 3 weeks. In the combination arms, durvalumab was continued until the disease progression or intolerable toxicity. The primary endpoint (OS) of this study was achieved in the experimental arm, comparing durvalumab with chemotherapy alone, showing an OS of 13 months vs. 10.3 months. However, no significant difference was found in PFS of 5.1 vs. 5.4 months. The ORR was 68%, with a slight benefit for the combination therapy (58%). No evidence of excessive toxicity was found, with the incidence of treatment-related grade III-IV adverse events were 46% and 52% in the experimental and control group respectively. The most common adverse events were neutropenia and anemia. Incidence of irAEs was 20% vs. 3% in the durvalumab arm and control arm, in which hyperthyroidism and hypothyroidism were the most common.

#### (3) Pembrolizumab

Pembrolizumab is an anti-PD-1 monoclonal antibody, which has shown promising clinical activity in the phase Ib, open-label, multi-cohort clinical trials (KEYNOTE-028) for the treatment of patients with recurrent/metastatic SCLC. Patients with previously treated recurrent/metastatic SCLC and PD-L1-positive expression received pembrolizumab monotherapy and achieved an ORR of 33.3% with median OS of 9.7 months.^139^ In a phase II, open-label and multi-cohort study (KEYNOTE-158), patients with recurrent/metastatic SCLC receiving pembrolizumab monotherapy had an ORR of 18.7% and an OS of 8.7 months. In both studies, pembrolizumab showed a favorable safety profile. Due to the results from these two studies, pembrolizumab has been approved for the treatment of metastatic SCLC patients.

To investigate further, a phase III, double-blind and placebo-controlled study (KEYNOTE-604) was designed to compare pembrolizumab plus etoposide and platinum (EP) with placebo combined with EP as first-line therapy for patients with previously untreated extensive-stage SCLC.^132^ Primary endpoints were PFS and OS. In the final analysis, Pembrolizumab plus EP significantly improved PFS, and the 12-month PFS rates were 13.6% vs. 3.1% respectively. However, OS was numerically longer in the experimental arm (10.8 vs. 9.7 months), but there was no statistical difference. The ORR for pembrolizumab-PE and PE arms was 70.6% vs. 61.8%, respectively. These data also support the therapeutic activity of pembrolizumab in patients with ES-SCLC.

#### (4) Nivolumab

Nivolumab as monotherapy or in combination has been investigated in multiple clinical trials in different SCLC settings. FDA has approved nivolumab as a third-line or later-line treatment for patients with advanced SCLC, regardless of the expression status of PD-L1. In CheckMate 032, the efficacy of nivolumab monotherapy versus nivolumab combined with ipilimumab was evaluated in patients with progressive disease after platinum-based chemotherapy. In total, 216 patients were enrolled in this study, and the primary endpoint was ORR.^140^ ORR was 10% with nivolumab alone (NIVO3), 23% with nivolumab 1 mg/kg plus ipilimumab 3 mg/kg (NIVO3+IPI1) and 19% with nivolumab 3 mg/kg plus ipilimumab 1 mg/kg (NIVO1+IPI3). There was no relationship between therapeutic responses and lines of therapy or PD-L1 expression. There were 13% Grade III–IV adverse events reported in NIVO3 arm, 30% in NIVO1+IPI3 arm and 19% in NIVO3+IPI1 arm. There was a promising survival rate, with 2-year OS rate of 26% and 14% for nivolumab plus ipilimumab and nivolumab alone.^141^ This study not only supports the additional study of nivolumab plus ipilimumab in the treatment of SCLC but also demonstrates the potential benefit of combined immunotherapy in this disease.

A phase III trial (CheckMate 451) was conducted to assess nivolumab monotherapy and nivolumab combined with ipilimumab as maintenance therapy following first-line chemotherapy in patients with ES-SCLC.^142^ Overall, this trial included 834 patients with a minimum follow-up of 8.9 months. However, there was no significant prolongation of OS in nivolumab plus ipilimumab group versus placebo (9.2 vs. 9.6 months). Moreover, severe AEs were more common in the combination group with more grade 3-4 AEs (52% vs. 12%). Subgroup analysis showed that patients with TMB over 13 mutations per megabase had an OS benefit from nivolumab plus ipilimumab.

#### (5) Camrelizumab

Camrelizumab is a humanized anti-PD-1 monoclonal IgG4 antibody. PASSION (NCT03417895) was a phase II clinical trial of camrelizumab combined with apatinib in patients with ES-SCLC after prior treatment with platinum-based chemotherapy.^143^ The primary endpoints were ORR and safety. Eligible patients were assigned randomly (1:1:1) to three cohorts: camrelizumab 200 mg every two weeks plus apatinib 375 mg orally once daily (QD cohort), or camrelizumab 200 mg every 2 weeks plus apatinib 375 mg d1-5 qw (qw cohort), or camrelizumab 200 mg every 2 weeks plus apatinib 375 mg d1-7 q2w (q2w cohort). In all, 59 patients were enrolled and 47 were in the QD cohort. In the QD cohort, the confirmed ORR was 34.0% (95% CI 20.9-49.3), with a median PFS of 3.6 months (95% CI 1.9-4.6), and median OS of 8.4 months (95% CI 4.7-12.3). In this trial, camrelizumab in combination with apatinib has shown an acceptable toxicity profile. The grade III-IV TRAEs were reported in 43 (72.9%) of all 59 patients, with hypertension (25.4%), decreased platelet count (13.6%), and hand-foot syndrome (13.6%) were the most common side effects. Camrelizumab plus apatinib has shown potential antitumor activity and acceptable toxicity in patients with ES-SCLC after platinum-based chemotherapy, and this phase II trial requires further clinical studies.

#### (6) Tislelizumab

Tislelizumab (BGB-A317), a humanized monoclonal IgG4 antibody with higher specificity and affinity for PD-1, has been investigated in hematological cancers and advanced solid tumors.^144^ Tislelizumab has a low affinity for Fc receptor, which may lead to improved anticancer efficacy.^145^ The multicenter, open-label, phase II clinical trial (NCT03432598) was designed to assess the safety, efficacy and potential predictive biomarkers of tislelizumab combined with chemotherapy for patients with advanced lung cancer as first-line treatment.^146^ In all the 54 patients, 17 patients with SCLC were enrolled, and the primary endpoint was ORR. All patients accepted tislelizumab 200 mg combined with 4-6 cycles of platinum doublet, and the SCLC cohort received etoposide plus platinum. Confirmed ORRs of SCLC was 77%, median PFS was 6.9 months, and median OS was not reached for all cohorts except SCLC cohort (15.6 months). The disease control rate (DCR) and median OS of tislelizumab combined with doublet chemotherapy were superior to the previous studies numerically,^133,147^ although direct comparisons and larger sample size cohorts are still needed to confirm these results. Three phase III studies have been initiated to evaluate tislelizumab combined with chemotherapy as the first-line treatment for different histology types of advanced lung cancers (NCT03594747, NCT03663205, and NCT04005716).

### B7-H3

The B7 family molecules, as an important class of negative costimulatory immune checkpoint molecules, has been uncovered through the in-depth study of tumor microenvironments, and has attracted attention in recent years. B7 family molecules could bind to their receptors, and then regulate early T-cell activation negatively, damage the immune function of T cells and induce the inactivation of infiltrating T cells in tumor microenvironments, which cause the tumor cells to escape immune surveillance and promote tumor development.^148^ B7-H3, also known as CD276 or B7RP-2, is a member of the B7 ligand family, and appears to be the “right” target for antibody-based immunotherapy.^149^ The ADC approach has been tested utilizing MGC018 (humanized B7-H3 mAb with a cleavable linker-duocarmycin payload, MacroGenics) which delivers duocarmycin to tumors. A phase I/II trial is assessing its safety alone or in combination with an anti-PD-1 mAb in B7-H3-expressing solid tumors (NCT03729596). DS-7300a (Daiichi Sankyo), a B7-H3-specific mAb conjugated to four topoisomerase I inhibitor particles is being tested in a phase I/II trial (NCT04145622).^150^

### Immune evasion and resistance

On the basis of the high burden of tumor mutation in SCLC cells, strong T-cell responses can be predicted. In fact, SCLC patients with paraneoplastic neurological syndromes show higher immune activity and better prognosis.^151^ Immunotherapy, which enhances the activity of T cells against cancer cells, has shown some benefits in patients with SCLC.^140,141^ However, only about 15% of patients with SCLC have response to T-cell checkpoint blockade.^133^ The limited efficacy of immunotherapies in SCLC is associated with a number of mechanisms, such as the major histocompatibility complex (MHC) class I molecules have low expression on the surface of SCLC cells.^152–155^ In addition, immuno-suppressive immune cells such as regulatory T cells, present in the tumor microenvironment of SCLC and may promote the immune evasion further.^156,157^ Moreover, SCLC cells can secrete neuropeptides to suppress the antigen-presenting cells.^158^ The development of chimeric antigen receptor-expressing T (CAR-T) cells and activation of macrophages may be helpful to improve the efficacy of T cells currently.^159,160^ A novel proposed tumor classification system called TIME (Tumor Immunity in the MicroEnvironment) has been adopted to predict response to immunotherapy.^161^

**Fig. 3:**
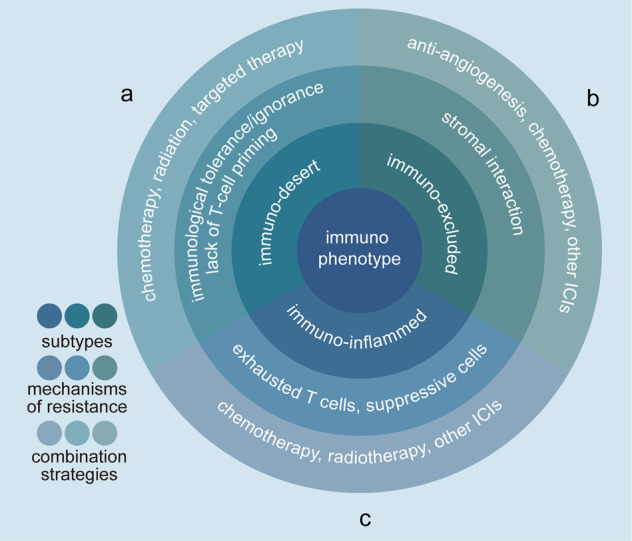
Potential combination strategies of ICIs in order to overcome resistance based on the immune phenotype. Antitumor immunity can be classified into three main phenotypes: the immuno-desert tumor (**a**), the immuno-excluded tumor (**b**) and the immuno-inflamed tumor (**c**). Each of this phenotype is associated with multiple mechanisms of resistance to immunotherapy. In order to overcome resistance to single-agent CPI, combination strategies have been suggested. The most promising ones comprise the combination with another ICIs, cytotoxic chemotherapy, radiation, antiangiogenesis or targeted therapies

SCLC is a challenging disease, because it tends to be diagnosed at an advanced stage, progress and metastasize rapidly, and acquire resistance to conventional chemotherapy and radiotherapy. The most significant mechanisms for drug resistance in SCLC are epigenetic modulation, an increase in tumor neoantigen burden, T-cell exhaustion, signaling pathways, transcriptional signature, and the microbiome. Immunotherapy has been implemented as a standard in the clinical routine, with durable response rates in patients with SCLC. However, it has been observed that most patients don’t present response to treatment initially or relapse after a short period of response.^162^ Immunotherapy resistance can be categorized into primary resistance and secondary (acquired) resistance.^163^ The former is shown to prevent the infiltration of immune cells into the tumor microenvironment (TME), while the latter has relationship with the components of TME except for tumor cells.^164^ It has been reported that, primary resistance to ICIs in patients with lung cancer accounts for 7-27% of first-line treatment and 20-44% of second-line treatment.^165,166^ It is also a dynamic process in which the immune/tumor cell balance determines the therapeutic response, and the TME provides a space for tumor development with chronic inflammatory and immunosuppression.^167–169^ In addition, different resistance mechanisms are depending on the immune phenotype. Immuno-desert tumors are characterized by immunological tolerance, ignorance or lack of T-cell priming. Immuno-excluded tumors can escape stromal factors because of the mechanical barriers, immune-suppressive chemokines and vascular factors. In immuno-inflamed tumors, all of these mechanisms come together.^169,170^ Resistance to ICIs is an emerging problem in clinical routine as a result of the increasing numbers of patients treated with immunotherapy. Although the results of immunotherapy combination trials are promising and led to approvals, future clinical trials should take into account the mechanisms of resistance and select patients according to the specific host immune microenvironment (Fig. 3).

## Signaling pathways targeting angiogenesis

The significance of angiogenesis has been supported by a large body of literature in tumor progression.^171^ The processes of angiogenesis are initiated in the early stage of tumorigenesis and remain activated throughout the course of the disease.^172^ Some components of the vascular endothelial growth factor (VEGF) signaling pathway have been confirmed to be overexpressed in SCLC, which is associated with poor outcomes.^173^ Hypoxia-inducible factor-1a (HIF-1a) has been demonstrated that it can promote tumor growth and angiogenesis, and proposed to be a potential therapeutic target for the treatment of SCLC.^174^ In addition, imatinib has been found to downregulate the expression of VEGF in SCLC cell lines by blocking c-kit inducible HIF-1a,^175^ however even in c-kit positive tumors, this approach has not been shown to be effective.^176^ Agents targeting angiogenesis have shown mixed results in SCLC, several anti-VEGF agents have failed to demonstrate improved outcomes in clinical trials with SCLC patients.^177^

### Bevacizumab

Bevacizumab is a humanized monoclonal antibody inhibiting angiogenesis, which has been approved for treatment in a variety of malignancies, including SCLC. In a large phase III study, bevacizumab combined with standard chemotherapy has shown to improve PFS but not OS.^178^ Similarly, in the SALUTE trial, there were 102 patients randomly assigned to standard chemotherapy plus bevacizumab or placebo. After 4 cycles of chemotherapy, patients received bevacizumab or placebo as maintenance therapy until progressive disease. Median PFS in the bevacizumab group was higher than the placebo group (5.5 vs. 4.4 months). However, Median OS showed no statistical difference between the two groups (9.4 months for bevacizumab group vs 10.9 months for placebo groups).^179^ In order to improve the outcomes of topotecan as second-line treatment for SCLC, 50 patients were enrolled in a phase II single-arm trial and treated with oral topotecan plus bevacizumab. The 3-month PFS, as the primary endpoint of the study, reached 65%, which was higher than the historical control of 50%, but did not reach the pre-defined criteria of clinically significant improvement.^180^ Notably, Bevacizumab combined with chemoradiotherapy has relationship with a high incidence of tracheoesophageal fistulae in LS-SCLC, medication warnings in bevacizumab is labeling as a result.

### Sunitinib

Sunitinib (SU11248) is an oral multi-target small-molecule receptor tyrosine kinase inhibitor (TKI) and inhibits angiogenesis through VEGFR and PDGFR-β.^181^ It was evaluated as maintenance therapy after chemotherapy in a randomized phase II trial (CALGB 30504).^182^ In the trial, 95 patients who had completed 4-6 cycles of chemotherapy without progression were randomly assigned 1:1 to sunitinib or placebo. The median PFS was 3.7 vs 2.1 months for sunitinib and placebo group, whereas the median OS was 9.0 months for sunitinib and 6.9 months for placebo, respectively. These results indicated that sunitinib may be beneficial in patients with extensive-stage SCLC. However, other studies have not shown a significant benefit from this drug.^183,184^ In summary, the substantial toxicity and insignificant benefits clinically indicate that further evaluation of the drug in SCLC is not warranted.

### Anlotinib

Anlotinib is also a small-molecule TKI targeting VEGFR, FGFR, PDGFR, and C-KIT.^185^ It was evaluated in a phase II study for the efficacy as a third or subsequent line treatment compared with placebo in SCLC. The median PFS was 3.9 months versus 0.7 months for anlotinib and placebo group. The DCR was also significant higher in the anlotinib group (71.6% vs. 13.2%). Moreover, there was a significant superiority of anlotinib with an OS of 7.3 months vs. 4.4 months in the placebo group.^186^ Based on these results, another phase II clinical trial (ALTER 1202) was conducted to evaluate the combination of anlotinib with standard chemotherapy in the first-line treatment for SCLC patients.^187^ Patients received anlotinib combined with conventional chemotherapy for 4-6 cycles, and then maintenance therapy if responded. Preliminary results of the trial showed that the median PFS was 9.6 months and the response rate was 77.7%. Data of phase III study is still awaited to determine its role in the first-line treatment of ES-SCLC patients. Several clinical trials are currently ongoing to combine anlotinib with anti-PD-1 inhibitors such as sintilimab. Interim analysis of a phase I trial has shown encouraging ORR of 77.3% and DCR of 100% in pre-treated ES-SCLC patients with an acceptable toxicity profile.^188^

### Apatinib

As a small-molecule inhibitor of VEGFR-2 tyrosine kinase, Apatinib blocks the transmission of the signaling pathway of VEGF/VEGFR-2, and has shown efficacy against a variety of cancers, including SCLC.^189^ A retrospective study showed that apatinib displayed efficiency in patients with ES-SCLC after failure of more than two prior chemotherapy regimens.^190^ A prospective, single-arm, phase II study was conducted to evaluate the safety and efficacy of apatinib in combination with etoposide taken orally as a third or subsequent line of treatment for ES-SCLC. Fifty-three patients with ES-SCLC who progressed after 2-3 prior lines of treatment were enrolled to receive apatinib and etoposide capsules, with PFS as the primary endpoint.^191^ The results showed that the median PFS was 3.0 months and the median OS was 5.0 months, respectively. The ORR was 20.8% and the DCR was 90.6%. Meanwhile, the 6-months OS rate was 40.1%, and 18.4% for 12 months, respectively. These findings suggest that apatinib combined with etoposide could potentially to be a third- or further-line therapy for patients with ES-SCLC. However, this study was a single-arm trial without a control group for comparison, and election bias cannot be ruled out.

## Potential therapeutic targets under investigation

### CD155 and T-cell immunoglobulin and ITIM domain (TIGIT)

The T-cell immunoglobulin and ITIM domain (TIGIT) is highly expressed on activated T cells and can inhibit T-cell functions after binding to its ligand CD155 on antigen-presenting cells.^192^ TIGIT, CD96, and CD112R compete as co‑inhibitors for their ligands (CD155 and CD112).^193^ The inhibition of TIGIT plays a critical role in different stages of tumor development.^194^ Anti‑TIGIT, anti‑CD96 or anti‑CD112R have all shown anti‑tumor effect in preclinical settings.^195–197^ However, the immunomodulatory role of CD155 in tumor microenvironment is rarely studied. Previous study has reported that higher expression levels of PD‑L1 and CD155 on CD8+ TILs and tumor cells were independent prognostic factors for better prognosis in SCLC patients.^198^

Recently, there are many clinical trials are ongoing to evaluate the safety and the efficacy of anti-TIGIT mAb either as a monotherapy or combined with ICIs for the treatment of various cancers. The CITYSCAPE trial, a phase II study evaluating the efficacy of tiragolumab plus atezolizumab in PD-L1-positive NSCLC, presented a significant ORR improvement (37% vs. 21%) as well as PFS improvement (5.6 vs. 3.9 months) for the combination group. More importantly, patients with high PD-L1 expression in the combination group had an ORR of 66% compared with 24% of the atezolizumab group.^199^ The phase III trial (SKYSCRAPER-01) is currently investigating tiragolumab plus atezolizumab in patients with newly diagnosed NSCLC and PD-L1 expression of at least 50%. Early-phase trials are also exploring tiragolumab in SCLC.^200^

### Cellular therapy

Adoptive cellular therapy (ACT), aiming to ignite tumor-specific immune response, has shown prospect in various malignancies by its ability to be trafficked into “poorly inflamed” tumors.^201^ CAR-T therapy, considered as a variation on ACT, has recently achieved remarkable success in the treatment of hematologic malignancies, including acute lymphoblastic leukemia.^202^

Those cell surface molecules, with great expression on the surface of SCLC cells, have been preferentially considered as potential therapeutic targets, such as CD47 and CD56.^159,203^ Delta-like ligand 3 (DLL3), which is an inhibitory Notch pathway ligand in over 80% of SCLC, upregulating and overexpressing in high-grade neuroendocrine tumors, whereas there is almost no expression in normal tissue, becomes an attractive therapeutic target.^86,204^ A phase I clinical trial adopting CAR-T cells with specificity targeting DLL3 (AMG 119) is currently ongoing (NCT03392064). An analogous approach targeting DLL3, applying the bispecific T-cell engager (BiTE) AMG 757, is currently underway (NCT03319940).^160,205^ CAR-T therapies have been tested recently in various solid tumors, and is expected to be investigated further in SCLC.

### Antitumor vaccines

Vaccines are proposed to act by stimulating the immune system to recognize and target specific tumor-derived neoantigens. Vaccines function by introducing a tumor antigen or pool of antigens with immunostimulant vehicles that sensitize the host’s T cells and drive them toward a cytotoxic response against the abnormal cells.^206^ Therapeutic vaccines are biological response modifiers that activate the immune system’s ability to target tumor-derived neoantigens in the tumor by introducing a tumor antigen or pool of candidate antigens. Antitumor vaccines may be an effective method to eliminate residual disease and prolong survival.^207^

BEC2 is an anti-idiotypic monoclonal antibody that mimics disialoganglioside with three glycosyl groups (GD3), which is expressed in most SCLC cell lines as a glycosphingolipid antigen. The data from Grant et al. suggested that immunization with BEC2 plus bacille Calmette-Guerin (BCG) for 15 SCLC patients with partial of complete response after completing standard therapy achieved a considerable longer median relapse-free survival, with mild adverse effects of mild fever and a local skin reaction.^208,209^ Subsequently a phase III clinical trial of BEC2/BCG vaccination in responding patients with LS-SCLC was conducted in 515 patients. However, there was no improvement observed in PFS or OS in these patients. This may be related to the humoral response developed only in one-third of the patients, and the GD3 expression was not evaluated for or stratified.^210^

Another anti-idiotypic monoclonal antibody against GM3 ganglioside, named 1E10, was applied in a phase I trial conducted for SCLC patients who achieved partial or complete response after receiving chemotherapy and/or radiotherapy. A prolonged survival was observed, including two patients with ES-SCLC who survived beyond 20 months and three patients with LS-SCLC who survived beyond 40 months.^211^ Nonetheless, further development of 1E10, which is also called racotumomab, has not been pursued in SCLC. A further study was conducted including another vaccine targeting fucosyl-alpha1-2Galbeta1-3GalNAcbeta1-4(NeuAcalpha2-3) Galbeta1-4Glcbeta1-1Cer (Fuc-GM1), another ganglioside expressed on the SCLC cell surface.^212^ The vaccine is a synthetic of Fucosyl GM1 conjugated to keyhole limpet hemocyanin, and the study showed a robust antibody response.^213^ Polysialic acid (polySA) is a polymer side chain that binds to the neural cell adhesion molecule, and is commonly expressed on the surface of nearly all human SCLC cells.^214^ However, a subsequent trial of NP-PolySA-KLH vaccine detected peripheral neuropathy and ataxia in patients vaccinated with high doses.^215^ The development of the vaccine has been limited because one of the 18 patients in the follow-up trial developed self-limited grade 3 ataxia.

As the majority of SCLC patients indicate a lack functional p53, it becomes an attractive target due to the direct involvement in the malignant transformation of tumors.^216^ Therefore, a study on an antitumor vaccine consisted of dendritic cells transduced with the full-length wild-type P53 gene was conducted for patients with ES-SCLC. In the study, most of the patients had progressive disease, but those received salvage chemotherapy immediately following the vaccination presented a high rate of ORR. The study provides an emerging paradigm wherein the use of the vaccine in combination with chemotherapy, rather than as a separate modality. A subsequent phase II trial was conducted in patients with recurrent SCLC.^217^ The primary endpoint was ORR of the salvage chemotherapy with paclitaxel after immunization with the p53 vaccine. No benefit in survival was observed between arms. The vaccine failed to increase ORRs as the second-line therapy in the study, but combinations with other chemotherapy agents are considerable.

Neoantigen-based peptide or RNA vaccines have shown encouraging efficacy in glioblastoma and melanoma.^218,219^ A pilot study (ChiCTR-ONC-16009100, NCT02956551) was designed to treat advanced lung cancer with a personalized neoantigen peptide-pulsed autologous dendritic cell (DC) vaccine (Neo-DCVac).^220^ In this trial, a total of 12 patients were enrolled, including 2 patients with SCLC. Results showed that all treatment-related adverse events (TRAE) were of grade I-II, with ORR of 25% and DCR of 75%, median PFS of 5.5 months, and median OS of 7.9 months, respectively. The study demonstrated that Neo-DCVac was practicable, safe and efficacious. Moreover, Neo-DCVac could give rise to antigen-specific T-cell responses and generate antitumor immune response.

In the past years, studies were conducted on vaccines targeting tumor antigens shared by SCLC patients, yet outcomes were disappointing. Vaccinating patients with individual tumor mutations may become the follow-up choices (Table 3).^221^

**Table 3:** Prior researches of antitumor vaccines for small-cell lung cancer cells or patients

Vaccine name	Target	Adjuvant	Usage and dosage	Targeted patients	Survival	Adverse effects	Phase of trial
Anti-Idiotypic Antibody BEC2 Plus BCG	The ganglioside GD3 expressed on the surface of most SCLC tumors	Bacillus Calmette-Gue ´rin (BCG)	Five intradermal immunizations consisting of 2.5 mg of BEC2 plus BCG (2×10^7^ CFU at first immunization and the dose reduced during the subsequent immunizations) over a 10-week period	Patients achieved PR or CR after initial therapy and without subsequent relapse or progression	OS: 20.5 m	Mild fever and a local skin reaction	/
				patients with limited-disease SCLC after a major response to chemotherapy and chest radiation.	OS: 16.4 m VS 14.3 m	Local skin toxicity, flu-like symptoms, lethargy	III
1E10 vaccine	Gangliosides having the N-glycolylated sialic acid (NeuGc), sulphated glycolipids and antigens present in lung tumors	Not mentioned	Four biweekly intradermal vaccinations with 2 mg of aluminum hydroxide‑precipitated 1E10 MAb, then other six doses at 28‑day intervals, patients maintained a good performance status were reimmunized.	SCLC patients achieved PR of CR after receiving chemotherapy and/or radiotherapy	2 with ES-SCLC survived beyond 20 months and 3 with LS-SCLC survived beyond 40 months	Local reaction at the injection site, fever, arthralgia, and cephalea	/
Synthetic Fucosyl GM1 Conjugated to Keyhole Limpet Hemocyanin	The ganglioside fucosyl GM1	QS-21	Three dose levels of fucosyl GM1-KLH conjugate at 30, 10, and 3 µg, with QS-21 100 µg. Vaccinations intradermally on weeks 1, 2, 3,4, 8, and 16.	SCLC, limited or extensive stage, who had completed initial therapy with chemotherapy (and radiation if needed) at least 4, but not more than 12 weeks previously		Injection site reaction, peripheral sensory neuropathy, myalgias, flu-like symptoms, arthralgias, fever, or chills, cough, fatigue	/
NP-polySA-KLH	Polysialic acid (polySA)	QS-21	Vaccinations intradermally with either 10 or 3 μg of NP-polySA-KLH and mixed with 100 μg of QS-21 at weeks 1, 2, 3, 4, 8, and 16.	SCLC patients who had completed initial treatment and had no evidence of disease progression	Median OS 22.9 m	1 patient with self-limited grade 3 ataxia of unclear etiology	/
TP53-transfected dendritic cell-based vaccine (Ad.p53-DC)	P53	Not mentioned	Arm A (observation) arm B (vaccine alone) arm C (vaccine plus all-trans-retinoic acid). Vaccinations intradermally every 2 weeks (three times), and all patients were to receive paclitaxel at progression.	Patients with extensive-stage disease	No statistically significant difference	Fatigue, headache	II

### Oncolytic virotherapy

Oncolytic virus therapy is the treatment of malignant tumors with replicating viruses.^222^ Historical evidence suggested that certain viral infections could lead to spontaneous remission of hematologic malignancies and solid tumors. Therefore, oncolytic virus therapy could be a potential cancer treatment.^223^ In recent times, only a few clinical trials with oncolytic virus were conducted in patients with SCLC (Table 4). The hypothesis was tested that tumors with a primarily high expression level of Coxsackie adenovirus receptor may be more suitable for oncolytic therapy in SCLC.^224^ A recombinant Coxsackievirus B3 (CVB3), which is powerful in destroying TP53/RB1-mutant SCLC, was generated to reduce the toxicity toward normal tissues. However, the downside is that the CVB3 genome as an RNA virus is less stable than that of DNA viruses.^225^ Seneca Valley Virus (SVV) is a novel naturally occurring oncolytic RNA virus of the Picornaviridae family, with potent and selective tropism for SCLC, as well as other neuroendocrine cancer cell types. SVV showed oncolytic activity in SCLC NCI-H446 cell line.^226^ SVV has been evaluated in a phase I clinical trial of advanced solid tumors with neuroendocrine features.^227^ Of all the five patients with SCLC involved, one patient with chemo-refractory SCLC remained PFS for 10 months after treatment, and is still alive for more than 3 years.

**Table 4:** Prior researches of oncolytic virus for small-cell lung cancer cells or patients

Virus name	Features	Usage and dosage	Patients/cell line characteristics	Survival	Response	Adverse effects	Phase of trial
microRNA-modified Coxsackievirus B3	Tumor-suppressive miR-145/miR- 143 target sequences into the viral genome	Injected intraperitoneally with a single dose of 1×10^8^ PFU in a volume of 100 µL for 14 days	TP53/RB1-mutant small-cell lung cancer	/	Powerful in destroying TP53/RB1-mutant SCLC, with a negligible toxicity	/	Animal experiment (xenograft model)
MYXV	A modified oncolytic myxoma virus (MYXV), Leporipoxvirus, lethal to European rabbit strains but nonpathogenic for other mammals	Injections of vMyx-FLuc (5 × 10^7^ FFU in 50 μl)	Tissue samples from 26 patients with SCLC	/	High efficiency for tumor-specific cytotoxicity in small-cell lung cancer efficient tumor-specific viral replication and cytotoxicity associated with induction of immune cell infiltration	/	Animal experiment
Seneca Valley Virus (SVV-001)	An concolytic picornavirus with tropism for neuroendocrine cancer cell types	Intravenous for patients at 10^7^ for the SCLC patients enrolled	Five patients with SCLC	One patient experienced disease stabilization persisted for ten months and remains alive after more than 3 years	Posttreatment neutralizing antibody titers in small-cell carcinoma patients were higher	flu-like symptoms Including pyrexia, malaise, myalgias, and arthralgias	I
Seneca Valley Virus (NTX-010)	An concolytic picornavirus with tropism for neuroendocrine cancer cell types	Intravenously as a 1-h infusion in 100 mL normal saline as a single dose. Patients in the NTX-010 arm received 10^11^ vp/kg	Patients with ES-SCLC who did not progress after completion of first-line chemotherapy	Median PFS: 1.7 m VS 1.7 m for treatment and placebo arms, and PFS was shorter in patients with detectable virus versus not detected	No improvement in PFS compared to placebo	flu-like symptoms, diarrhea, fatigue, thrombocytopenia	II

Subsequently, a randomized phase II study of the SVV (NTX-010) was conducted in patients with ES-SCLC.^228^ In this trial, patients were randomized to SVV or placebo within 12 weeks of chemotherapy if not progressed after more than four cycles of platinum-based chemotherapy. Results showed that disease response rate and OS were not improved with NTX-010 treatment after chemotherapy. Unexpectedly, detectable NTX-010 in the blood at day 7 or 14 after treatment was associated with shorter PFS. At the pre-specified interim analysis, no significant advance in PFS was observed between patients who received NTX-010 compared to placebo and the trial was closed for futility. Besides, Poirier et al. assessed the efficacy of SVV-001 in primary heterotransplant mouse models of SCLC. The result indicated that the ratio of NEUROD1 to ASCL1 may act as a predictive biomarker for the efficacy of SVV-001.^228^ Kellish et al. studied a modified oncolytic myxoma virus (MYXV) with ability to infect and replicate in tumor cells.^229,230^ The study demonstrated the safety of intrapulmonary MYXV in an immunocompetent mouse model with SCLC and the viral replication and cytotoxicity in specific tumor cells. In addition, the oncolytic activity delivery with induction of immune cell infiltration indicated the potential to increase the immunogenicity of tumor and improve the response rates to immunotherapy.

## Conclusion and perspectives

SCLC remains a cancer with poor prognosis, despite good initial response to systemic therapies. Most patients develop advanced disease and rapidly develop resistance to treatment and subsequently succumb to their disease. While targeted therapies have dramatically changed the way for treating patients with NSCLC, there have been no comparable breakthroughs in patients with SCLC.

However, recent understanding of tumor biology through molecular and genetic studies has shown significant improvements and will potentially lead to major breakthroughs for patients with SCLC. In particular, comprehensive genomic, proteomic, and transcriptomic analyses have identified several novel targets in SCLC including PARP1, BCL-2, WEE1, EZH2, and DLL3. Rapid advances in the field of immune-oncology have also emerged as a new treatment option, including ICIs, antitumor vaccines and oncolytic virus. Moreover, the immune microenvironment of SCLC appears to differ from other solid tumors and offer new avenues of therapeutic strategies. In addition, there are increasing efforts in incorporating predictive biomarkers for target-matched therapies and multiple clinical trials are currently exploring this approach.

Importantly a multidisciplinary, collaborative, and inter-institutional approach is warranted to make rapid progress in this deadly disease. This includes broad access to clinical trials for patients with SCLC, sampling and long-term storage of tumor material including blood samples, access to molecular pathology labs, and collaboration with pharma and biotech companies.
